# L. P. Yandell, Jr., M. D.

**Published:** 1868

**Authors:** 


### L. P. YANDELL, JR., M. D.

With this number of the Journal we publish the last of a series of letters from this able and talented gentleman. We thank him in our own behalf, and also that of our subscribers. We wish him the fame to which he aspires, and to which his learning and talents entitle him.

He has returned to Louisville and resumed the practice of medicine.

